# The Role of NMNAT2/SARM1 in Neuropathy Development

**DOI:** 10.3390/biology13010061

**Published:** 2024-01-22

**Authors:** Olga Tarasiuk, Laura Molteni, Alessio Malacrida, Gabriella Nicolini

**Affiliations:** Experimental Neurology Unit, School of Medicine and Surgery, University of Milano-Bicocca, 20900 Monza, Italy; laura.molteni@unimib.it (L.M.); alessio.malacrida@unimib.it (A.M.); gabriella.nicolini@unimib.it (G.N.)

**Keywords:** NMNAT2, SARM1, CIPN, neuropathy, inhibitors

## Abstract

**Simple Summary:**

Chemotherapy-induced peripheral neuropathy (CIPN) is a common side effect of various cancer chemotherapy treatments, often leading to dose reduction or treatment discontinuation. The lack of a comprehensive understanding of its underlying mechanisms has hindered the development of effective CIPN treatments. Recent investigations focusing on axon degeneration mechanisms have identified NMNAT2 and SARM1 as key mediators of injury-induced axonal degeneration. In this review, our objective is to examine various studies shedding light on the interplay between NMNAT2 and SARM1 proteins and their roles in the progression of CIPN. We discuss three main aspects of the NMNAT2/SARM1 interplay: (1) the balance between NMNAT2 and SARM1 and their direct impact on NAD+ synthesis at the cellular level and axonal translocation; (2) the regulation by NMNAT2 and SARM1 of crucial downstream players implicated in neuronal cell death; (3) the role of SARM1 in activating immune cells, potentially contributing to the development of neuroinflammation. Additionally, we review the existing literature that explores the involvement of NMNAT2 and SARM1 in CIPN development and provide a summary of information on available inhibitors as potential therapeutic options.

**Abstract:**

Chemotherapy-induced peripheral neuropathy (CIPN) commonly arises as a side effect of diverse cancer chemotherapy treatments. This condition presents symptoms such as numbness, tingling, and altered sensation in patients, often accompanied by neuropathic pain. Pathologically, CIPN is characterized by an intensive “dying-back” axonopathy, starting at the intra-epidermal sensory innervations and advancing retrogradely. The lack of comprehensive understanding regarding its underlying mechanisms explains the absence of effective treatments for CIPN. Recent investigations into axon degeneration mechanisms have pinpointed nicotinamide mononucleotide adenylyltransferase 2 (NMNAT2) and sterile alpha and TIR motif-containing 1 protein (SARM1) as pivotal mediators of injury-induced axonal degeneration. In this review, we aim to explore various studies shedding light on the interplay between NMNAT2 and SARM1 proteins and their roles in the progression of CIPN.

## 1. Introduction

NMNAT, a highly conserved nicotinamide adenine dinucleotide (NAD+) synthase, plays a crucial role in cell survival by controlling the compartment-specific levels of NAD+ and influencing various cellular processes such as protein balance, cell differentiation, DNA repair, maintenance of neurons, and more. Three NMNAT isoenzymes exist: NMNAT1 which is localized in the nucleus and is widely distributed in tissues; NMNAT2 which is localized in the cytoplasm and Golgi apparatus and mainly exists in the nervous system, heart, and skeletal muscle; NMNAT3 localized in mitochondria and expressed in the spleen and lungs. NMNAT1 stands out as the most prevalent among the isoforms, demonstrating also the highest catalytic efficiency. Additionally, NMNAT1 exhibits about a fourfold greater specificity for nicotinamide mononucleotide (NMN) compared to nicotinic acid mononucleotide (NaMN). NMNAT2 displays the lowest efficiency, utilizing NAMN and NMN with similar effectiveness as NMNAT3, which is also typically found in lower abundance compared to NMNAT1 [1]. However, whether the substrate specificity of this isoform varies based on its subcellular localization remains unexplored.

In particular, NMNAT2, which predominates in the brain, attracts scientific attention for its possible role in neuroprotection. NMNAT2 has two fundamental biological roles: (1) it acts as a NAD+ synthetase by transferring the adenosyl portion of adenosine triphosphate (ATP) to NMN, resulting in the reversible synthesis of NAD+—this function is crucial for neuroprotection and is strongly linked to the development and outcome of malignant tumors [2,3]; (2) moreover, NMNAT2 acts as a molecular chaperone by activating the distinctive ATP site situated in its C-terminal region. This activation results in the creation of a complex with heat shock protein 90 (HSP90) [4]. The formation of this complex enhances the refoldase activity of NMNAT2, aiding neurons in overcoming protein unfolding and aggregation. Consequently, this process helps alleviate protein-induced stress and preserves the overall health of neurons [5]. NMNAT2 has a notable impact on diverse medical conditions, encompassing neurodegenerative diseases and malignant tumors. Apart from its crucial protective role in neurodegenerative diseases, NMNAT2 exhibits high expression in various solid tumors, playing a significant role in tumor occurrence and progression [6]. NAD+ produced by NMNAT2 acts as a redox coenzyme for numerous dehydrogenases in energy metabolism and as a co-substrate for various enzymes that regulate diverse cellular processes. NAD+ acts as a critical substrate for various enzymes, such as PARPs, CD38/157 ectoenzymes, and sirtuins (histone deacetylases), which are involved in DNA repair, apoptosis, calcium signaling, and transcriptional regulation—processes integral to tumor growth and survival [7,8]. It is well established that cancer cells exhibit a higher demand for NAD+ compared to normal cells. Specifically, NMNAT2 levels increase in colorectal cancer, showing a positive correlation with tumor invasiveness and stage [9]. Elevated levels of NMNAT2 are also observed in ovarian cancer cells. In this context, NMNAT2 was found to enhance the activity of PARP16, which, through mono ADP-ribosylation of ribosomal proteins, maintains proteostasis during accelerated cell proliferation. Deletion of NMNAT2 leads to protein aggregation, consequently reducing the growth of cancer cells [10]. Depending on the tissue-specific NAD(P)ase that NMNAT2 (through NAD+ production) regulates, it can have a distinct functional outcome.

SARM1 serves as a key player in programmed axon death, inducing the destruction of NAD+ when activated. In mammals, SARM1 is abundantly present in neurons, is localized both in cell bodies and axons, and can be found in association with mitochondria [11]. Due to its prominent presence in the nervous system, research on SARM1 primarily focuses on neuron degeneration. However, SARM1 is also observable in other tissues, particularly in macrophages and T cells. SARM1-ko effectively prevents axon degeneration [12,13,14]. However, even a partial reduction provides significant protection [15]. Interestingly, SARM1 also plays a role in innate immunity [16]. Its composition incorporates a Toll/interleukin-1 receptor (TIR) domain, a characteristic frequently identified in Toll-like receptors (TLRs) of the innate immune system.

The NMNAT2/NAD+/SARM1 interplay has a crucial role in Wallerian degeneration, a programmed form of subcellular death that facilitates axon degeneration. Decreased levels of NMNAT2, triggered by physical injury or pathological stimuli, activate SARM1, leading to axonal degeneration and the onset of neurodegenerative diseases and peripheral neuropathy [6]. It is however important to discuss that NMNAT2/SARM1 regulation is a complex process and is not completely understood. Here, we discuss NMNAT2/SARM1 regulation and its role in chemotherapy-induced peripheral neuropathy focusing on three main aspects, namely: (1) NMNAT2/SARM1 balance and its direct role on NAD+ synthesis at cellular level and axonal translocation; (2) NMNAT2/SARM1 regulation of important downstream players that can be involved in neuronal cell death; (3) SARM1 and its role in the immune cells activation that can play a role in neuroinflammation development.

## 2. NMNAT2/SARM1 and NAD+ Synthesis

**Figure 1:**
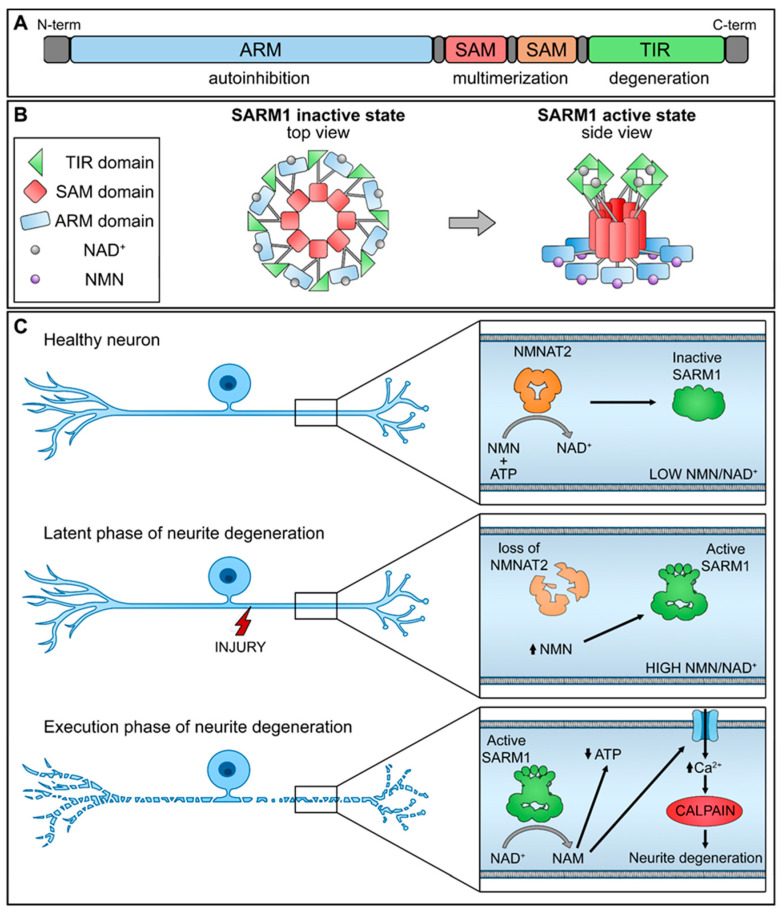
Schematic representation of NMNAT2/SARM1 regulation mechanism. (**A**) SARM1 domains; (**B**) SARM1 active and inactive state; (**C**) interplay of NMNAT2/SARM1 during neurite degeneration. (up arrow-increase; down arrow-decrease).

SARM1 is composed of distinct domains, including an N-terminal allosteric regulatory armadillo repeat (ARM) domain, two tandem sterile alpha motif (SAM) domains, and a C-terminal catalytic TIR domain (Figure 1A). The presence of the ARM domain is crucial for exerting an autoinhibitory function, effectively preventing the dimerization of TIR domains required for SARM1 enzymatic activity. Recent structural investigations have revealed the inactivated state of SARM1, wherein it forms an octomeric ring structure (Figure 1B) [17].

NMNAT2 exerts regulatory control over the activation of SARM1 by competitively binding to the allosteric sites within the N-terminal ARM domain of SARM1, utilizing both NAD+ and its precursor NMN. In functioning nerve cells, an elevated concentration of NAD+ engages and stabilizes the ARM domain, promoting its interaction with the TIR domain and efficiently suppressing the NAD+ hydrolase activity. Nevertheless, a decline in NMNAT2 levels, whether triggered by physical injury or pathological stimuli, compromises the conversion of NMN into NAD+, leading to diminished NAD+ levels within the axons. As a result, the NAD+ bound to the ARM dissociates, diminishing its binding affinity with TIR and reinstating the NAD+ hydrolase activity within the TIR domain.

This sequence of events leads to metabolic disruption and axonal rupture (Figure 1C). Changes in the NMN-NAD+ ratio, achieved through elevating NMN levels or diminishing NAD+ levels, can equally trigger the NADase activity of SARM1. NAD+ and NMN engage in direct competition to control the enzymatic activity of SARM1 [18]. Therefore, the equilibrium between the protective factor NMNAT2 and the destabilizing molecule SARM1 is crucial for preserving axonal integrity.

Moreover, it is important to point out that NMNAT2, produced in the cell body, afterward is translocated to axons and is trafficked bidirectionally [19,20]. Associated with transport vesicles, it becomes susceptible to ubiquitination and subsequent degradation by the proteasome, resulting in reduced stability [19,20]. Disruption or injury to axonal transport impairs the delivery of NMNAT2 to distal axons, leading to its depletion and subsequent degeneration [19,21]. To demonstrate the vesicular nature of the transport organelle, NMNAT2 was demonstrated to co-migrate with Golgi and synaptic vesicle markers, indicating that through Golgi-derived vesicles NMNAT2 enters into axons. In particular, it was found that the palmitoylation site C164/165 is essential for the vesicular axonal transport of NMNAT2 [22,23]. At first, there was a hypothesis suggesting that a vesicle-bound fraction of NMNAT2 would be shielded from degradation during transport, given the substantial time it takes to reach the distal ends of long axons. Nevertheless, in contrast to this presumption, Milde et al. showed that cytosolic NMNAT2 mutants, which lacked palmitoylation and vesicle association, exhibited a longer protein half-life compared to the vesicle-bound wild-type form [19]. Given the critical role of palmitoylation in NMNAT2 membrane association, the enzymes involved in both NMNAT2 palmitoylation and depalmitoylation are potentially significant in influencing NMNAT2 subcellular localization and turnover.

Surprisingly, it has been shown that the neuroprotective effect of NMNAT2 in axons is enhanced when it loses its association with vesicles. This impact is, in part, attributable to the prolonged half-life of cytosolic NMNAT2 variants.

Research utilizing fluorescently tagged NMNAT2 expressed in mouse peripheral nerves revealed substantial stabilization following the removal of regions responsible for vesicle attachment [24]. One could propose that the extension of the half-life is, in part, a result of decreased ubiquitination levels in the cytosolic forms, leading to enhanced axon protection in primary culture neurites [19]. Additional exploration revealed that the connection with vesicle membranes facilitated the ubiquitination and turnover of NMNAT2, as evidenced by the reversal of alterations when cytosolic mutants were redirected to vesicle membranes using various methods [25].

## 3. NMNAT2/SARM1 Downstream Pathways Regulation

Several studies propose that maintaining NMNAT2/SARM1 balance is crucial for regulating various pathways and highlights the significance of some downstream players involved in neuronal survival and axon degeneration.

The study conducted on various model organisms has elucidated the involvement of SARM1 in controlling mitogen-activated protein kinase (MAPKs) signaling pathways that are relevant for cell life, neuronal plasticity, and innate immunity spanning across both neurons and glial cells [26]. The involvement of MAPKs in axon degeneration has been well established, as inhibiting the MAPK cascade, including dual leucine zipper kinase (DLK) and c-Jun N-terminal kinase (JNK), has shown to provide protection similar to SARM1 knockout following axonal injury [27]. Nevertheless, the recent study suggests that the interplay between MAPK signaling and SARM1 activity may be more complex than initially thought. The role of MAPKs in degeneration downstream of SARM1 has been a matter of discussion, but recent research indicates that they might serve alternative signaling functions. It has been shown that both the NADase activity of SARM and a cascade involving cacophony (Cac)/SARM/MAPK are essential for an initial phase of axon transport blockage after axotomy [28]. Moreover, Yang and co-researchers illustrated that injury triggers the activation of the MAPK signaling pathway in a SARM1-dependent manner. This results in the subsequent disturbance of axonal energy homeostasis, leading to ATP depletion before the activation of calpains and the breakdown of axonal structures. The MAPK cascade signifies an early degenerative response, and SARM1 is crucial for its activation following injury [27]. Various papers emphasize the significance of calpain in the SARM1-activated pathway. It is proposed that taxane-based chemotherapy-induced neuropathy activates calpains, either directly or indirectly. Upstream NMNAT2 activity reduction and NMN accumulation trigger SARM1 activation. It is believed that calpain activation takes place downstream of SARM1 [29]. Specific calpain inhibitors have been shown to prevent cisplatin (CDDP) [30] and paclitaxel (PTX)-induced peripheral neuropathy [31,32].

However, the relationship between SARM1 activation and MAPK needs to be better examined. Notably, it was found that although MAPK signaling is essential for injury-induced NAD+ depletion and axonal degeneration, it does not play a role in NAD+ depletion or axon degeneration triggered by activated SARM1 [33]. This controversially implies that MAPK signaling might function upstream of SARM1, facilitating its activation in response to injury. Inhibition of MAPK signaling leads to a reduction in the turnover of both NMNAT2 and the microtubule-destabilizing factor SCG10, leading to elevated levels of these survival factors. Additionally, elevated levels of NMNAT2 are necessary for the protective effects against axon degeneration brought about by inhibiting MAPK signaling. This revised understanding places MAPK signaling at the beginning of the chain of events leading to axon degeneration, where it regulates the turnover of axon survival factors and activates SARM1 [33].

Additionally, it is important to notice, several investigations have indicated primarily the involvement of JNK signaling in the progression of axon degeneration. Stress-related signaling mediated by DLK and downstream JNKs has been recognized as activated in various aspects of the axonal injury response [34,35]. These kinases have been observed to activate in damaged axons shortly after nerve injury [27,36,37]. It is suggested that JNK functions downstream of SARM1, given that genetic disruption of all three JNK isoforms (or both MKK4/7) can counteract the degeneration caused by the ectopic activation of SARM1’s TIR domains [27]. However, this aspect requires further investigation, as one study proposes an upstream pro-degenerative role for JNK, where JNK promotes the degradation of NMNAT2 [26]. Moreover, the three JNK kinase isoforms all have the capacity to phosphorylate SARM1. Phosphorylation of SARM1 activates its enzymatic activity, leading to subsequent neurodegeneration [38,39].

On the other hand, different noteworthy papers have established a connection between SARM1 activation and the production of downstream cyclic adenosine diphosphate (ADP)-ribose (cADPR) as an important player in axon degeneration. Recent findings have revealed that cADPR is responsible for inducing axonal calcium flux and degeneration in response to PTX. Genetic or pharmacological inhibitors of cADPR signaling have been shown to prevent axon degeneration and alleviate symptoms of allodynia induced by PTX without compromising the anti-neoplastic efficacy of the drug. These findings highlight cADPR as a calcium-modulating factor that promotes axon degeneration, thereby proposing cADPR signaling as a potential therapeutic target [40]. Moreover, it is suggested that cADPR may contribute to the pathophysiology of pain before degeneration is through the activation or sensitization of transient receptor potential (TRP) channels [41], which has been associated with pain, including mechanical allodynia induced by oxaliplatin (OXP) [42]. Moreover, cADPR was observed in both uninjured and injured human sensory neurons, showing the preservation of SARM1’s metabolic and degenerative roles across human and mouse neurons and is being suggested to be a biomarker of SARM1 enzyme activity [43].

## 4. NMNAT2/SARM1 Role in Immune System Stimulation

The involvement of glial cells in neuropathic pain is a well-established fact. Increasing evidence suggests that immune cells are not merely passive bystanders in the nervous system but active influencers in the onset and/or development of neuropathies. It is well demonstrated that axon injury leads to the recruitment of macrophages to the site of injury, a process orchestrated by different cell populations. Within 2–3 days post-injury, macrophages are the primary and most abundant cells infiltrating the injury site, attracted by factors released by repair Schwann cells. These macrophages, in turn, produce chemoattractants like CCL2, TNF-α, IL-1α, IL-1β [44], NGF, and nitric oxide (NO) [45] enhancing further macrophage infiltration.

**Figure 2:**
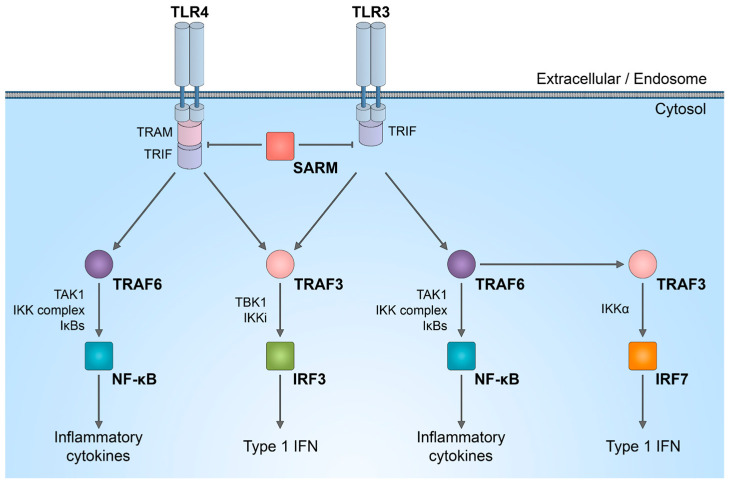
SARM1 role in innate immunity activation. Schematic representation of inhibitory effect of SARM1 in TLR signaling pathways.

It is important to discuss that SARM1 plays a role in innate immunity, participating in the regulation of TLR signaling, as well as the synthesis of cytokines and chemokines within neurons [46,47] and innate immune cells [48,49]. In complex in vivo models, immune response activated by SARM1 could potentially be linked to axon degeneration. While the enzymatic activity of SARM1 NADase has been intensively studied in neurons, its role in the immune system is not yet fully understood. SARM1 is one of the six adaptor proteins involved in TLR signaling pathways. TLRs serve as indispensable pattern recognition receptors, playing a pivotal role in the innate immune system. Upon stimulation of TLRs, multiple transcription factors, including nuclear factor (NF)-κB, interferon regulatory factor 3 (IRF3), IRF5, and IRF7, are triggered (Figure 2). Subsequently, these factors induce the expression of numerous genes that code for immunoregulatory molecules, including type I interferons, chemokines, and inflammatory cytokines.

The distinctive combination of three protein–protein interaction domains in SARM1 implies that, within the TLR adaptor family, SARM1 likely operates in a manner distinct from other adaptor molecules. There is a suggestion that in humans, SARM1 exerts a negative regulation on TRIF-dependent TLR3 and TLR4 signaling, leading to the inactivation of the NF-κB response [50]. Reduction in the natural SARM1 expression boosts TRIF-dependent cytokine and chemokine production. This establishes SARM1 as a targeted inhibitor of TRIF signaling and specific innate immune responses [50].

**Figure 3:**
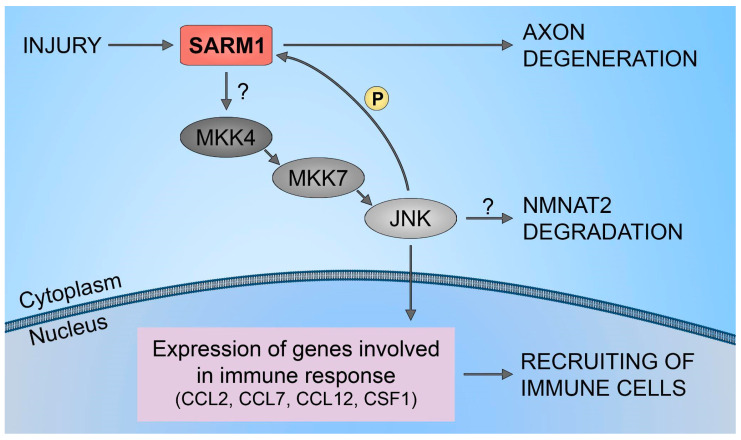
SARM1/JNK pathway. Schematic representation of SARM1 role in immune cells recruitment to injured neural tissues.

Furthermore, it has been demonstrated that SARM1 plays a crucial role in the upregulation of chemokines CCL2, CCL7, and CCL12, along with the cytokine CSF1. However, traumatic axonal injuries did not alter the expression levels of SARM1. Also, increasing the full-length SARM1 protein in cultured neurons did not influence the level of expression of CCL7, CCL2, CCL12, and CSF1. This implies that the neuronal immune response, orchestrated by SARM1, is unlikely to be governed by the levels of SARM1 protein expression. This mechanism is shown to be regulated through the SARM1-JNKs-cJun pathway (Figure 3). The results have identified JNK2 and JNK3 as essential signaling components downstream of SARM1 in the neuronal immune response to traumatic axonal injuries. Studies have demonstrated the rapid activation of JNK proteins in cultured neurons following traumatic axonal injuries. In neurons lacking JNK2 and JNK3 (JNK2−/−; JNK3−/−), the significant upregulation of CCL2, CCL7, CCL12, and CSF1 was noticeably suppressed compared to neurons with partial deficiency (JNK2+/−; JNK3+/−). This highlights the essential involvement of JNK2 and JNK3 in the immune response of neurons, indicating that blocking this pathway is an effective measure to impede the mobilization of immune cells to damaged neural tissues. Furthermore, inhibiting the SARM1-JNK pathway not only obstructs the recruitment of immune cells but also impedes the degeneration of axons [51].

Moreover, it is crucial to acknowledge that SARM1 is present in macrophages, playing a primary role in controlling the initial recruitment of transcription factors and RNA polymerase II to the CCL5 promoter. Research indicates the essential role of SARM1 in facilitating optimal CCL5 production upon TLR4 and TLR7 stimulation. Notably, the induction of TNF, IL-1β, CCL2, and CXCL10 genes occurs independently of SARM1. SARM1 is not a prerequisite for the TLR-induced activation of MAPKs or the transcription factors associated with CCL5 induction, including NF-κB and IFN regulatory factors. Additionally, SARM1 does not impact CCL5 mRNA stability or splicing [52].

Beyond TLR signaling, SARM1 has been noted to have other innate immune functions. Inflammasome activation during injury can lead to cytokine release or pyroptosis. In mice, it has been demonstrated that SARM1 serves as a negative regulator for NLRP3 inflammasome-dependent caspase-1 activation, leading to a decrease in the release of IL-1β. It has been shown that both the SAM and TIR domains of SARM1 are essential to initiate pyroptosis. After NLRP3 activation, SARM1 clusters at the mitochondria and facilitates mitochondrial depolarization (MDP), a crucial process for efficient pyroptosis. This sets it apart from other NLRP3 activators that induce cytokine release [53].

## 5. NMNAT2/SARM1 in Chemotherapy-Induced Neuropathy

As previously mentioned, an imbalance between NMNAT2 and SARM1 is associated with axon degeneration, particularly in the context of chemotherapy-induced neuropathy (Table 1). The in vitro study has demonstrated that preserving axonal NAD+ levels and inhibiting SARM1 can effectively prevent axon degeneration. Genetic removal of SARM1 significantly reduces vincristine (VCR) and bortezomib (BTZ)-induced axon degeneration, both in vitro and in vivo [54]. Specifically, exposure to VCR or BTZ leads to a reduction in axonal NMNAT2 levels. The activation of SARM1 occurs through the decrease in NMNAT2, whereas the study demonstrated that preserving a consistent NMNAT isoform in the axon offers strong protection against both VCR and BTZ-induced axon degeneration. SARM1 activation induces a swift and significant reduction in NAD+, leading to subsequent local metabolic collapse and axon degeneration. The prompt decrease in NAD+ within the axon following the administration of VCR and BTZ is facilitated by the activation of SARM1. This illustrates that SARM1 operates via the identical downstream mediator (NAD+) and is controlled by the immediate upstream regulator (NMNAT2). Moreover, it has been noted that the SARM1-induced decrease in NAD+ is specific to the axon, not the neuronal soma, indicating a selective activation of SARM1 in the axon. An important finding is that effectively addressing the fundamental axon degeneration program, either by sustaining NAD+ levels or inhibiting SARM1, entirely and enduringly halts axon degeneration induced by VCR and BTZ, both in laboratory settings and in living organisms [54].

Additionally, in vivo studies confirm that the absence of SARM1 gene expression counteracts the progression of acute painful neuropathies triggered by chemotherapeutics, such as OXP [41], VCR [14], PTX [54], and CDDP [30]. Nevertheless, in animal models, characterized by a complexity surpassing that of cell cultures, it has been demonstrated that the molecular pathway(s) of axon degeneration involving SARM1 may not universally apply to all instances of axon degeneration. It has been shown that absence of the SARM1 gene elicits exhibited resistance to PTX-induced distal axonal degeneration in CIPN. Partial preservation of thermal sensitivity and epidermal innervation was noted, although no impact was observed on the reduction in tail sensory nerve action potential amplitudes. This result contrasted with the neuroprotection seen in the presumed metabolic neuropathy model, where thermal sensitivity, tail sensory nerve action potential amplitude, and epidermal innervation were all preserved [55]. In the other study, the PTX mechanism of action in CIPN development is suggested to be associated with cADPR. Treatment with PTX leads to a notable increase in cADPR levels in distal axons, but not in cell bodies, while both NAD+ and NADP levels show slight decreases in both distal axons and cell bodies. These findings establish a correlation between the local buildup of cADPR in axons and PTX-induced axon degeneration [40]. The same finding was confirmed as well in vivo model, where inhibition of SARM1 has been shown to reduce the elevation of nerve cADPR levels and the release of plasma neurofilament light chain of injured sciatic nerves in vivo [56].

**Table 1:** SARM1 regulation related to CIPN.

Drug Class	Chemotherapy Drug	SARM1 Modulation	References
Vinca alkaloid	Vincristine	Absence of SARM1 gene expression block axonal degeneration induced by VCR. VCR reduces NMNAT2 levels activating SARM1 and leading to axon degeneration.	Geisler et al., 2016 [14] Geisler et al., 2019 [54]
Taxane	Paclitaxel	Mice lacking SARM1 gene are resistant to axonal degeneration induced by PTX. Pharmacological inhibition of SARM1. protects axons from PTX-induced degeneration.	Turkiew et al., 2017 [55] Bosanac et al., 2021 [57]
Proteasome inhibitor	Bortezomib	BTZ reduces NMNAT2 axonal levels leading to SARM1 activation.	Geisler et al., 2019 [54]
Platinum compounds	Cisplatin Oxaliplatin	CDDP activates SARM1 and calpain leading to axon degeneration. SARM1 deficiency confers resistance to OXP-induced neuropathies in mice.	Cetinkaya-Fisgin et al., 2020 [30] Gould et al., 2021 [41]

In another in vivo study, emphasis is placed on the downstream protein calpain in the SARM1 pathway. It is demonstrated that the activation of calpains is crucial for both neurotoxicity and the formation of DNA-platinum adducts in neurons. This is supported by the fact that a calpain inhibitor effectively blocked the formation of DNA-platinum adducts in neuronal cells treated with CDDP. Notably, Sarm1−/− mice treated with CDDP did not exhibit an increase in calpain activity in sciatic nerves. Moreover, these animals showed only a minimal increase in DNA-platinum adducts in the DRG compared to wild-type mice, which exhibited a significant increase. These data confirm that SARM1 and calpain interplay is necessary for progression of CDDP-induced neuropathy [30]. In another study, a more precise mechanism of calpain regulation has been suggested. Following axon damage, the levels of the calpain inhibitor calpastatin decrease. This reduction in calpastatin is a consequence of the actions of SARM1 and is probably due to an increase in cellular calcium levels. Once calpastatin’s inhibitory effect is lifted, calpains induce the breaking down of the axonal cytoskeleton. Chelating extracellular calcium or inhibition of calpain may counteract this process and prevent structural deterioration of axons. However, these interventions do not fully preserve the electrophysiological functionality of the axon. It is likely that the influx of calcium and the subsequent activation of calpain mark the final stages in the fragmentation of a metabolically inactive axon [56,58].

A significant discovery is that the overexpression of SARM1 does not trigger axon degeneration in the absence of injury caused by axotomy or vincristine treatment. These findings underscore the pivotal role of SARM1 in the process of axon degeneration, emphasizing that, even when overexpressed, SARM1 necessitates an injury signal to initiate axon degeneration. It is probable that the regulation of SARM1 occurs at the post-translation level. SARM1-dependent axon degeneration is unlikely to involve the transcription of new SARM1 molecules. This suggests two general potential processes: (1) SARM1 is expressed in uninjured neurons at levels sufficient to induce axon degeneration in response to injury without the need for de novo transcription; (2) further, SARM1 is activated after the axon is severed, and it triggers a localized self-destructive pathway within the axon. It is possible that SARM1 has an inherent ability to induce degeneration, contingent on its TIR domain. However, this capacity is suppressed in the full-length SARM1 protein, indicating that the complete SARM1 molecule is in a dormant state. The capability of SARM1 to initiate a pathway leading to cell death provides evidence that SARM1 is closely involved in the self-destructive mechanism of axons. This suggests that SARM1 might be present in axons in an inhibited state and gets activated after translation, possibly due to mechanisms triggered by injury [59].

The complexity of the in vivo model compared to the in vitro model is once again represented in the other publication where the impact of depleting NMNAT2 on cell viability was less pronounced following PTX treatment compared to VCR treatment, possibly due to differences in the mechanisms of action between these agents. It has been demonstrated that chemotherapeutic drugs can disrupt axonal transport both in vitro and in vivo. In vitro investigations demonstrated that the total lack of NMNAT2 in cultured cortical neurons compromised the neuronal maintenance mechanisms that guard against toxic effects induced by chemotherapeutic agents. The impact was more prominent with VCR, which modifies the structure of the cytoskeleton and disrupts microtubules, compared to PTX, which interferes with the cell cycle and stabilizes microtubules. However, contrary to the in vitro findings, in vivo studies do not provide evidence for NMNAT2’s protective role in chemotherapy-induced peripheral neuropathy.

It was demonstrated that a 50% decrease in NMNAT2 protein does not influence chemotherapy-induced peripheral neuropathic allodynia, as assessed through responsiveness to mechanical and cold stimulation [60].

Additionally, it is believed that the neuroprotective effect of NMNAT2 appears to be specific to this particular isoform. It is thought that the specific reduction in NMNAT2 alone can trigger Wallerian-like degeneration in uninjured axons, a process that cannot be prevented by endogenous NMNAT1 and NMNAT3. The most convincing evidence supporting the role of NMNAT2 in maintaining healthy axons comes from observations where the knock-down of NMNAT2 through siRNA leads to neurite degeneration even without any injury, occurring prior to any impact on neuronal survival. Endogenous NMNAT1 and NMNAT3 are unable to recompensate for NMNAT2 loss, likely due to strict compartmentalization. This disparity could arise from the varied contributions of each isoform to the overall baseline NMNAT activity in axons, with NMNAT1 and NMNAT3 offering protection only when they are overexpressed. The overexpression frequently leads to notable mislocalization, resulting in an inadvertent rise in effective NMNAT levels within the pertinent axonal regions. The loss of NMNAT2 could also potentially explain axon degeneration in diseases characterized by a “dying-back” pattern, as the swift turnover of NMNAT2 might impede its capacity to reach distal axons in ample amounts, particularly in situations where axonal transport is pathologically compromised or slows down in the course of normal aging [20].

## 6. SARM1 Inhibitors

As mentioned above, there are currently no available treatments specifically addressing disorders linked to axonal degeneration. Inhibition of SARM1 represents an attractive therapeutic target for treating various pathologies of axon degeneration, including peripheral neuropathy, traumatic brain injury, and neurodegenerative disorders. In recent years, several studies have identified and developed small-molecule inhibitors targeting the SARM1 pathway (Table 2).

Using a high throughput screening that utilizes a NAD+ analog that fluoresces upon the release of nicotinamide, five putative SARM1 inhibitor compounds that exhibit competitive or non-competitive modes of inhibition with up to 70% efficiency were identified [61]. Other competitive and non-competitive SARM1 inhibitors were also identified through a modified fluorescence assay that measures the production of cADPR, the hydrolysis product of NAD+. These compounds also showed strong protection against axon degeneration, even if toxicity was manifest for almost all tested compounds [62]. Recently, a novel category of powerful non-competitive SARM1 inhibitors, which form adducts, was discovered to provide neuroprotection in preclinical models of nerve injury and disease [63].

In early 2021, a series of isoquinoline inhibitors were identified and developed as small-molecule inhibitors of SARM1 [64]. In particular, the selective inhibitor 5-iodo-isoquinoline (subsequently called DSRM-3716) was shown not only to inhibit SARM1 enzymatic NADase activity, but also to protect axons degeneration in vitro, reproducing the axonal protective SARM1−/− phenotype. Moreover, DSRM-3716 allowed the recovery of axons already in the intermediate injury state. Later, this series of isoquinoline compounds were shown to undergo NAD+-dependent base-exchange reactions producing NAD+ mimetic SARM1 inhibitor 1AD [65].

**Table 2:** Representative list of SARM1 inhibitors.

Compound	Inhibition Mechanism	IC_50_	Reference
Phenazopyridine hydrochloride Doxycycline hydrochloride Nitroflurazone Berberine chloride Pyrithone zinc	Competitive Non-competitive Non-competitive Non-competitive Non-competitive	145 µM 145 µM 90 µM 140 µM 20 µM	Loring et al., 2020 [61]
TK106 TK138 TK210 TK222	Non-competitive Competitive Competitive Competitive	10.8 µM 2.9 µM 4.6 µM 3.2 µM	Khazma et al., 2022 [62]
NB-3 NB-7	Non-competitive Non-competitive	0.195 µM 0.025 µM	Bratkowski et al., 2022 [63]
DSRM-3716	Reversible	75 nM	Hughes et al., 2021 [64]
Isothiazole—Compound 4 Isothiazole—Compound 9 Isothiazole—Compound 10	Irreversible; possibly at C635 or C649	0.37 µM 0.16 µM 0.23 µM	Bosanac et al., 2021 [57]
dHNN	Irreversible; possibly at C311	2.4 µM	Li et al., 2021 [66]
Tryptoline acrylamide EV-99	Covalent; selective at C311	4.7 µM	Feldman et al., 2022 [67]

An innovative set of irreversible isothiazole SARM1 inhibitors was studied for their effectiveness in preserving axonal structure and function, both in laboratory settings and, for the first time, in living organisms [57]. A sequence of optimization procedures led to the development of an orally bioavailable compound suitable, thanks to its submicromolar potency, for chronic dosing in vivo.

Dehydronitrosonisodipine (dHNN), a derivative of nisoldipine, was also discovered as an irreversible SARM1 inhibitor [66]. This compound reacts with Cys311 in the ARM domain and blocks its NMN-activation, protecting axons from neurodegeneration. The same cysteine residue was shown to be targeted by a series of tryptoline acrylamides that inhibit the NADase activity of SARM1 and prevent neurite degeneration induced by VCR [67]. Moreover, it has been shown that nicotinic acid mononucleotide (NaMN), the deaminated form of NMN, competes with NMN for binding to the allosteric pocket of SARM1. This competition inhibits SARM1 activation, thereby inducing long-term protection of the axon [68].

## 7. Conclusions

CIPN stands as a prevalent side effect resulting from antineoplastic agents independent of their mode of action. Various pieces of evidence indicate the association of the NMNAT2 and SARM1 pathway in CIPN. In addition, SARM1 serves as a NAD+-depleting enzyme, it also plays a role in activating the innate immune system, both mechanisms of which could contribute to CIPN development and progression. Additionally, studies propose that it may not be NMNAT2 and SARM1 proteins that are crucial targets; instead, it is suggested that downstream players in different pathways significantly participate in axon degeneration and subsequent neuropathy development. This review comprehensively summarizes existing research efforts aiming to analyze the interplay between NMNAT2 and SARM1, shedding light on their mechanisms of action in the development of CIPN, as well as possible targets for its therapy. The identification of novel SARM1 inhibitors with diverse modes of action (non-competitive inhibition, competitive inhibition, and covalent inhibition) developed in the last 2 years looks promising and is worthy of further development.

## Data Availability

Not applicable.
